# Serratus Anterior and Latissimus Dorsi Muscle Activation in Hypopressive Exercises Performed in Open Versus Closed Kinetic Chain: A Cross-Sectional Study

**DOI:** 10.3390/muscles4030020

**Published:** 2025-06-23

**Authors:** Esther Hernández Rovira, Diego A. Alonso-Aubin, Dolors Cañabate Ortiz, Carlota Torrents Martín, Tamara Rial Rebullido

**Affiliations:** 1 Faculty of Education and Psychology, University of Girona, 17004 Girona, Spain; esther.rovira@udg.edu; 2 National Institute of Physical Education of Catalonia (INEFC), University of Lleida (UDL), 25192 Lleida, Spain; ctorrentsm@gencat.cat; 3 Strength Training and Neuromuscular Performance Research Group, Faculty of Health Sciences-HM Hospitals, University Camilo José Cela, Villanueva de la Caña, 28692 Madrid, Spain; diegoalexandre.alonso@ucjc.edu; 4 HM Hospitals Health Research Institute, 28015 Madrid, Spain; 5 Department of Health and Physical Education, Monmouth University, West Long Branch, NJ 07764, USA

**Keywords:** electromyography, breathing exercises, scapula, rehabilitation exercises

## Abstract

This study aimed to describe and compare the serratus anterior (SA) and latissimus dorsi (LD) muscle activity during six hypopressive exercise (HE) positions performed in open and closed kinetic chains. While previous studies analyzed abdominal and pelvic muscle activity during HE, research on scapular stabilizers like SA and LD remains underreported. Twenty-five healthy adults (mean age, 42.9 ± 8.4 years; BMI, 22.1 ± 2.4 kg/m^2^) with prior HE experience performed three open and three closed-chain HE positions. Surface electromyography recorded bilateral SA and LD activity, normalized to maximum voluntary isometric contraction (MVIC). SA showed greater activation than LD across all positions, with moderate activation levels (20–40% MVIC), while LD activation remained mild (<20% MVIC). Significant differences were found across positions and kinetic chain conditions. SA activation was higher during closed-chain standing (W = 41; *p* < 0.001; r = −0.74) and kneeling (W = 9; *p* < 0.001; r = −0.94), while LD activity increased significantly in the seated closed-chain position (left LD: W = 26; *p* < 0.001; r = −0.84; right LD: W = 20; *p* < 0.001; r = −0.87). These findings suggest body and kinetic chain positioning influence scapular muscle recruitment during HE. Further research is warranted to determine clinical applications.

## 1. Introduction

Hypopressive exercise (HE) has emerged as a postural and breathing-based rehabilitation exercise program used by therapists in the training of trunk stabilizing musculature such as the diaphragm, transverse abdominis, and pelvic floor muscles [1,2]. Although previous studies have explored the application of HE in the treatment of pelvic floor dysfunctions [2], more recently, it has been applied as a scapular stabilization training option for adults with non-specific low back pain [3,4] and shoulder dysfunction [5]. HE is often performed with the arms elevated and the thoracic cage expanded through a breath-holding maneuver, “abdominal vacuum” [6], suggesting a potential contribution of periscapular and trunk musculature.

Our previously published scoping review [1] highlighted how surface electromyography (sEMG) studies in HE have focused mainly on the abdominal wall and pelvic floor muscle activation, while scapular stabilization muscle involvement remains unexplored. In particular, the serratus anterior (SA) and latissimus dorsi (LD) may play important functional and respiratory roles in thoracic cage positioning during HE postures, specifically when arms are elevated or supported.

To date, only one electromyographic study on healthy adult females addressed muscle activation of accessory respiratory muscles, such as the scalenes, sternocleidomastoid, and SA during HE [7]. However, only two HE positions, standing and seated, were explored in this study, and it did not assess the LD [7]. While the SA has been previously identified as a key accessory breathing muscle [8], the LD’s breathing function is less emphasized [9]. Of note, the LD shares costal attachments with accessory inspiratory musculature and connects to the thoracolumbar fascia [10]. These anatomical features suggest that the LD could contribute to thoracic cage lift and trunk stability [9,11], especially under respiratory and isometric demands like those found during HE.

HE is commonly performed in supported closed kinetic chain positions with the assistance of a wall, yet no previous research has analyzed how the kinetic chain configuration can affect shoulder girdle muscle activation during HE. Closed kinetic chain exercises involving scapular protraction (i.e., push-up, plank, wall-press) have been reported as efficient in maintaining the optimal activation of the SA [12,13,14] and are commonly prescribed in the initial rehabilitation of shoulder dysfunctions to enable periscapular muscle stabilization while decreasing the involvement of prime shoulder movers [12,15]. A better understanding of SA and LD muscle patterns during HE in closed versus open kinetic chain can aid in informing exercise selection in early-stage rehabilitation.

Therefore, we aimed to describe sEMG activation levels of the SA and LD and compare differences while performing HE in diverse open and closed kinetic chain positions. Due to the periscapular stabilization and inspiratory functions of the SA and LD [9,11], we hypothesized that both muscles would be activated during HE and that closed kinetic chain positions would achieve greater muscle recruitment than open kinetic chain positions during HE. This study aims to address a main gap in the HE literature by assessing muscle activation patterns on commonly used HE variations used by therapists, which could better inform exercise selection and prescription.

## 2. Results

All participants completed study-related tests successfully, and no unexpected adverse events or injuries occurred during the study. Participants’ levels of physical activity were mostly high (44%) and moderate (52%), whereas only 4% accumulated low levels of physical activity. The average years of HE training practice were 3.83 (SD, 3.51), and the mean weekly training frequency was 2.24 days (SD, 1.69). A total of 24 participants’ dominant side was right, and only one participant’s dominant side was left. The demographic and anthropometric descriptive data of participants are presented in Table 1.

The analysis of muscle activation by exercises and kinetic chain condition revealed significant differences for the left SA (standing: W = 41; *p* < 0.001; r = −0.74; kneeling: W = 9; *p* < 0.001; r: −0.94; and seated: W = 9; *p* < 0.001; r = −0.94), the right SA (standing: W = 2; *p* < 0.001; r = −0.98; kneeling: W = 1; *p* < 0.001; r: −0.99; and seated: W = 19; *p* < 0.001; r = −0.88), the left LD (seated: W = 26; *p* < 0.001; r: −0.84), and the right LD (seated: W = 20; *p* < 0.001; r: −0.87). The descriptive sEMG values for each exercise and kinetic chain condition and the results of the comparison of muscle activation across different muscle groups, positions, and kinetic chains can be found in Table 2.

Across all exercises, SA activation was higher than LD. SA activation levels ranged from 20–40% of MVIC, characterized as moderate activity. LD activation levels were below 20% of MVIC in all conditions, indicating low activation levels.

The comparison between positions based on the kinetic chains revealed significant differences in open-chain left LD (χ^2^ = 8.24; *p* = 0.01) and closed-chain left SA (χ^2^ = 7.44; *p* = 0.02), right SA (χ^2^ = 10.3; *p* = 0.006), left LD (χ^2^ = 27; *p* < 0.001), and right LD (χ^2^ = 29.44; *p* < 0.001). In addition, comparisons between exercises revealed significant differences in open-chain left LD (kneeling vs. seated: DC = 3.03; *p* = 0.004) and closed-chain left SA (standing vs. seated: DC = 2.70; *p* = 0.009 and kneeling vs. seated: DC = 2.25; *p* = 0.02), right SA (standing vs. seated: DC = 3.26; *p* = 0.002 and kneeling vs. seated: DC = 2.80; *p* = 0.007), left LD (standing vs. seated: DC = 5.92; *p* < 0.001 and kneeling vs. seated: DC = 6.94; *p* < 0.001), and right LD (standing vs. seated: DC = 6.68; *p* < 0.001 and kneeling vs. seated: DC = 7.54; *p* < 0.001). All the results can be found in Table 3 and Table 4.

## 3. Discussion

This study aimed to explore the muscle activity of the SA and LD between six pairs of HE performed in closed and open kinetic chain positions. To the best of our knowledge, this is the first study to analyze the sEMG of LD activation across diverse HE positions. Additionally, no previous studies aimed to assess differences between HE in closed vs. open kinetic chains. Our findings partially support the initial hypothesis that HE performed in a closed kinetic chain elicits greater SA muscle activity than open-chain positions in most of the studied conditions, specifically in standing and kneeling positions. However, the seated position revealed greater left SA activation in the open-chain condition. These results suggest that muscle recruitment during HE is influenced not only by the kinetic chain but also by body support and posture.

Overall, the SA showed a higher percentage of activation than the LD for all HE positions. SA reached a moderate percentage of activation across analyzed positions, while LD muscle recruitment remained low (below 20% of MVIC). Digiovani et al. [16] noted that moderate activation levels (21–40%) of MVIC could be adequate for early-phase muscular training in a rehabilitation context. LD activation, although mild, showed significant increases in the seated position for both sides compared to standing or kneeling. These data suggest that the LD likely serves an accessory role during inspiration and scapular stabilization in HE. Similarly, Hardwick et al. [17] found minimal LD activity during a wall-press exercise in a standing position with forearms against the wall with 90 degrees of humeral elevation. The selection of the seated position could facilitate the postural demands or thoracic cage lift requirements of the LD. However, these low LD muscle activity thresholds and effect sizes might be of insignificant clinical impact and inadequate for muscular training purposes.

SA percentage of MVIC was characterized by moderate activation levels with higher muscular recruitment in HE, where the center of gravity is further from the floor, such as in the standing position. Of interest, SA activation was higher in closed-chain standing and kneeling compared to seated. Hardwick et al. [17] found significant differences in SA activation when a standing wall-press exercise was performed with a humeral elevation higher than 90 degrees as compared to 90 degrees elevation. Machado et al. [7] also reported higher levels of SA activation in the standing and seated HE positions when compared to the supine HE positions in a group of healthy adult females. These results underscore the influence of supported arm position and body position for HE used to engage the SA for scapular stabilization.

Previous studies have analyzed shoulder and trunk muscular recruitment (i.e., infraspinatus, deltoids, serratus anterior, trapezius, erector spinae, and external oblique) with diverse closed kinetic chain exercises such as the wall-press [13], which is similar to the HE performed against the wall in our study. However, in HE, specific breathing maneuvers are added to the body position, which could result in differences in muscle activation. The sEMG data collection was conducted during the abdominal vacuum maneuver of HE, which is performed while keeping the thoracic cage high and ribs wide [6]. Research has demonstrated that LD muscle recruitment rises with inspiratory load and pressure generated [11]. Inspiratory muscle fatigue affects the activity of the LD [18] and the SA [9]. While the LD is active during a deep inspiration (inspiratory volume close to maximum vital capacity), it also assists in keeping the chest high [11]. However, the LD muscle activation during all HE positions in our study was less than 21% of MVIC.

Previous sEMG studies with HE have primarily focused on the pelvic floor and anterior abdominal muscles assessed in the supine, quadruped, prone plank, and standing positions [2,19,20]. In a group of healthy adult females, Ithamar et al. [19] used sEMG to evaluate abdominal wall and pelvic floor musculature activation during HE performed in the standing, quadruped, and supine positions. This study found significant differences between selected body positions, specifically in the standing position compared to supine [19], and Quiroz et al. [20] compared the sEMG activity of the rectus abdominis, transverse, and external oblique muscles during a prone plank exercise versus a prone plank exercise performed with the HE breathing technique. They reported that the addition of HE breathing to the prone plank exercise activated the transverse abdominus, internal oblique, and external oblique muscles to a greater degree than the traditional prone plank, while activation of the rectus abdominis was reduced [20]. Of interest, a voluntary abdominal contraction performed during closed kinetic chain exercises has been shown to activate a greater proportion of the SA [21]. The SA and LD, through costal attachments, are interdigitated to the costal attachments of the external oblique abdominis LD [8,10]. The superficial lamina of the thoracolumbar fascia is derived in large part from the aponeurosis of the LD [10]. The previously demonstrated activation of the external oblique during HE [19] could be a contributory mechanism for the SA and LD activation levels described in our study.

### 3.1. Limitations

Although statistically significant differences were found for kinetic chain and body positions, several comparisons yielded large effect sizes while others yielded low effect sizes, which may not translate into substantial functional outcomes for healthy individuals with experience in HE. Thus, this data should not be generalized to those with clinical conditions, including shoulder pathologies, and with different weight statuses. Future studies analyzing muscle activation during HE in specific groups, including novices or adults suffering from shoulder dysfunction or lumbar pain, are warranted. Additionally, sEMG was analyzed during isometric positions, which are commonly practiced in HE programs and generate a more reliable sEMG measurement. However, this limits the generalization of results to dynamic HE or other exercises of a similar nature. Additional studies using real-time dynamic analysis could complement our findings. Also, only two back muscle groups were analyzed. Future studies should evaluate EMG activation of the back and shoulder musculature during distinct positions such as quadruped, supine, standing, and in stable and unstable positions. A total of 84% of our sample were female, which limited the exploration of sex-based muscle activation differences. The potential sex-based muscle activation differences could affect the interpretation of the present results.

### 3.2. Future Perspectives

This study was designed to provide preliminary evidence on levels of activation of the SA and LD muscles during open and closed kinetic chain HE positions in healthy participants experienced in the technique. Although our findings revealed significant differences between HE positions, with large effect sizes for the SA, the small to moderate effects for LD limit the translation of results into clinical applications. Despite the limitations previously mentioned, these findings inform the selection and progression of HE for early-stage rehabilitation phases where scapular stabilization might be a therapeutic goal. Closed-chain standing or kneeling may be preferred for targeting moderate SA activation, while seated closed-chain may facilitate introductory LD activation.

In our study, body position influenced sEMG activation, with the standing and kneeling positions enabling better activation of the SA and the seated position for the LD. This information can better aid therapists and practitioners in the selection of standing HE positions in closed-chain for scapular stabilization rehabilitation. Closed kinetic chain HE could be useful in early phases of rehabilitation, for patients whose pain or limited range of motion might not tolerate high-load scapular exercises. These exercises could also be considered as part of a breathing-targeted program.

Additional research is warranted to determine the impact and clinical usefulness of HE for clinical conditions involving periscapular conditions. Future lines of research may include the assessment of dynamic sEMG in populations with shoulder or low back conditions, as well as an analysis of additional periscapular muscles such as the trapezius or intercostals. Lastly, sex-based and training status differences should be further explored.

## 4. Materials and Methods

### 4.1. Design

This study was a prospective cross-sectional study that used a repeated-measures, within-subject design. We used the checklist for reporting and critically appraising studies using EMG (CEDE-Check) as a comprehensive checklist for reporting the sEMG methodology used in this study [22] (see Supplementary Table S1).

### 4.2. Participants

A priori power analysis was conducted using G*Power 3.1 to estimate the required sample size. Although the statistical analyses performed in the study were non-parametric (Wilcoxon signed-rank and Kruskal–Wallis tests), a repeated-measures ANOVA model with two within-subject factors (Kinetic chain: open-chain and closed-chain × Exercise: standing, kneeling, and seated) was used in G*Power 3.1 as a standard approximation, assuming a medium effect size (f = 0.50), a significance level of α = 0.05, a desired power of 0.80, and a correlation among repeated measures of 0.5. This analysis indicated that a minimum of 12 participants were required to detect statistically significant effects. A total of 24 participants were ultimately included, providing adequate power to detect medium or larger effects. This approximation is commonly used when non-parametric tests are applied, in order to ensure methodological transparency and reproducibility [23,24] and aligns with the thresholds used in the present analysis for interpreting non-parametric effect sizes.

The study was conducted between December 2023 and February 2024. Recruitment of participants was made through physical therapy clinics from the Girona area (Spain) and by word of mouth. Inclusion criteria for participating in this study were: (a) to be between 20 to 60 years of age; (b) to have a minimum practice experience with HE of 12 weeks; and (c) to not have any musculoskeletal pain and/or dysfunctions in the shoulder or back regions. The exclusion criteria were: (a) any contraindication to performing the HE programs (hypertension, pregnancy, pulmonary obstructive dysfunctions) or (b) any injury that did not allow the completion of the HE. Before commencing the study, the participants were informed about data collection procedures and the intervention program. The study was approved by the Research Ethics and Biosafety Committee of the University of Girona (project number: CEBRU0046-23). All participants gave written informed consent to participate in the study.

### 4.3. Procedures

Data were collected in a physical therapy clinic. Before the testing session, height and body mass were measured using a precision stadiometer and balance (SECA 700). Participants underwent a familiarization session with the same evaluator to ensure understanding and execution of the HE intervention. Participants were also introduced to sEMG before all testing. The abbreviated format of the International Physical Activity Questionnaire (IPAQ) was used to characterize participants’ physical activity levels [25]. The intensity combined with the time spent on each activity was used to calculate the IPAQ summative score (MET min/week). The level of physical activity classification followed the criteria available in Craig et al. [25], which classifies physical activity levels as low, moderate, and high.

During the second session, testing was performed. Electromyographic data was collected bilaterally from the SA and LD using the mDurance^®^ system (mDurance Solutions SL, Granada, Spain) previously validated for this purpose [26]. This is a portable, wireless, and lightweight (30 g) sEMG system that consists of a bipolar sEMG Shimmer3 sensor composed of two sEMG channels (Realtime Technologies Ltd., Dublin, Ireland). The two sEMG channels in each Shimmer sensor have a sampling rate of 1024 Hz. Shimmer uses an 8.4 kHz bandwidth, a 24 bit EMG signal resolution, and an overall 100–10,000 V/V amplification. Pre-gelled Ag/AgCl electrodes with a 10 mm diameter and a 20 mm interelectrode spacing were utilized [26]. A total of 6 solid hydrogel adhesive electrodes of 30 mm size (DORMO^®^, Telic Group SL, Barcelona, Spain) and a band were used to hold the sensors. Two electrodes were used for each muscle and side. The electromyographic data was collected via Bluetooth from the Shimmer unit to the mDurance 2.4.0 (Android) mobile application using a digital tablet (Samsung, Suwon, South Korea) and a cloud service that stores the sEMG signals. The raw electromyographic data was digitally filtered using a fourth-order Butterworth bandpass bandwidth filter with a cut-off frequency at 20–450 Hz, and the signal was smoothed using a window size of 0.025 s root mean square to represent the amplitude values of the EMG [26].

Participants were asked to bring comfortable clothing that did not interfere with the placement of the electrodes. The sEMG testing followed the recommendations for the surface electromyography for the non-invasive assessment of muscles (SENIAM) guidelines [27]. The skin was shaved, scrubbed, and cleaned with 96° isopropyl alcohol (Micraderm, Celra, Girona, Spain), and allowed to dry to diminish skin impedance. The same evaluator tested all participants and was blinded to data analysis. Participants were seated with their shoulders in 90-degree abduction for bilateral electrode placement. For the SA, electrodes were positioned vertically along the axillary midline at the level of the sixth and eighth ribs in the direction of the muscle fiber, maintaining a distance of 2 cm between electrodes for adequate signal capture. For the LD, electrodes were placed lateral to thoracic vertebra 9, below the lower tip of the scapula, and over the muscular belly. The reference or ground electrode was placed on a superficial bony surface and distal to the electrodes, such as the iliac crest.

To calculate the maximal sEMG activity of each muscle and normalize the amplitude of the signal during HE, participants performed three maximal voluntary isometric contractions (MVIC) against manual resistance. Participants underwent 5 min of familiarization with the MVIC assessment before testing. For the SA, the participant was placed in a seated position with scapular rotation upwards and the shoulder at 125°. For the LD, the participant was positioned in the prone position, and an upward arm extension was requested. The evaluator applied manual resistance against the movement for 5 s for 3 repetitions with a 30 s rest between repetitions for each muscle group. Verbal encouragement was provided, such as “push as hard as you can” and “keep pushing”. The mean value of muscle activity (microvolts) was calculated over 10 s, and the average of the times in the different muscles and participants was used as the value of the analysis [28].

### 4.4. Intervention

**Figure 1:**
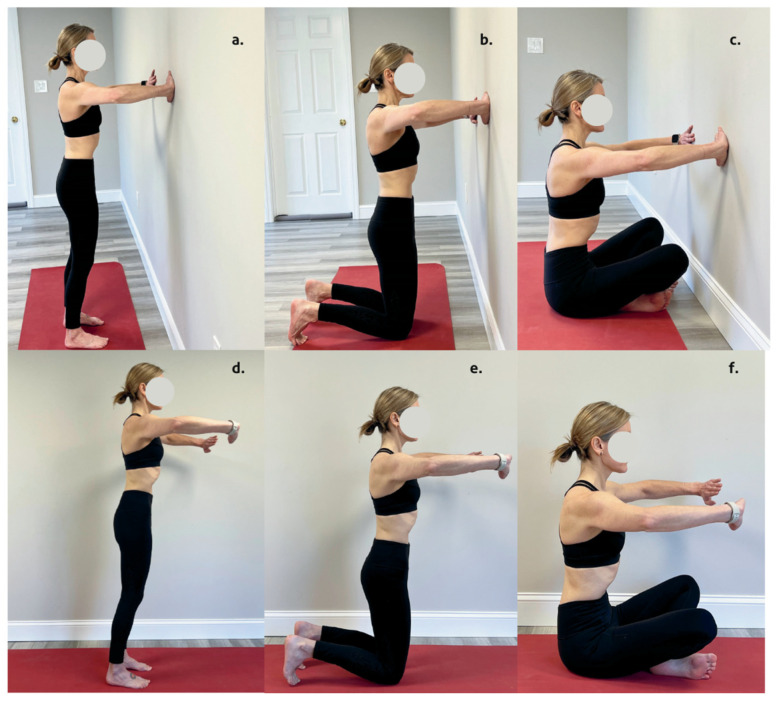
Protocol of HE analyzed (**a**) standing closed-chain; (**b**) kneeling closed-chain; (**c**) seated closed-chain; (**d**) standing open-chain; (**e**) kneeling open-chain; and (**f**) seated open-chain.

The HE was standardized and supervised by the same evaluator who had 7 years of experience teaching HE. Participants were barefoot and wore comfortable clothes. The HE was performed respecting the postural fundamentals described elsewhere [3,6]: (a) neutral pelvis and axial elongation; (b) dorsiflexion of the ankles for sitting, and kneeling positions; (c) knee bending; (d) scapular adduction of the shoulder girdle muscles; (e) three breathing cycles with lateral rib breathing and slow, deep exhalations (maximum inhalation and exhalation); (f) maintenance of breathing after expansion of the rib cage (abdominal vacuum phase). Instructions based on the postural fundamentals were provided during each position as a means of feedback and encouragement. The protocol consisted of 6 distinct positions: (a) standing open-chain; (b) kneeling open-chain; (c) sitting open-chain; (d) standing closed-chain; (e) kneeling closed-chain; and (f) sitting closed-chain. Exercise order was randomized for each participant, and positions were all performed on a mat or against the wall during closed kinetic chain positions. Figure 1 illustrates the analyzed positions. The selection of the HE positions was based on their beginner level and on being common positions identified in our scoping review of the HE literature [1]. Additionally, isometric arm and body positions were selected to minimize possible variations at the electrode site. All HE arm positions were maintained at 90 degrees of shoulder elevation at the scapular plane with the same degree of elbow flexion for all positions. The standardization of the body stance of the closed kinetic chain HE was based on the distance of the arm extended at 90 degrees of elevation. A total of 3 repetitions of each exercise were performed with 3 min of recovery between exercises. Breathing was cued with the following instructions: inhale for 2 s, exhale for 4 s, breath-hold while performing an abdominal vacuum for 10 s. Our previously described technical description of the abdominal vacuum served as a foundation for the protocol used in this study [6].

### 4.5. Statistical Analysis

Descriptive characteristics were calculated, and results were presented as means and standard deviations (SD), as well as medians. Normality of all variables was assessed using the Shapiro–Wilk test. Levene’s test was used to verify the homogeneity of the analyzed variables. The effect of the chains in each exercise (open-chain vs. closed-chain) was analyzed using the Wilcoxon signed-rank test, and the rank biserial correlation was used to determine the effect size, interpreted as follows: small >0.1 to 0.3; medium >0.3 to 0.50; and large >0.50 to 1.00. To assess differences between kinetic chains based on the exercises, a non-parametric repeated-measures ANOVA (Kruskal–Wallis test) was used, and the Durbin–Watson test was employed for pairwise comparisons between exercises. Statistical significance was set at *p* ≤ 0.05. All statistical analyses were performed using JASP (JASP Team, version 0.17.3 [Computer Software], Amsterdam, The Netherlands). Additionally, %MVIC was classified as very high (≥60%), high (41–60%), moderate (21–40%), or low (0–20%) according to previous research [16].

## Figures and Tables

**Table 1:** Baseline descriptive characteristics of participants (n = 25).

		Confidence Interval at 95%		
Characteristics	Mean (SD)	Lower Limit	Upper Limit	Median
Age (years)	42.9 (8.13)	39.56	46.28	45
Height (m)	1.67 (0.07)	1.64	1.70	1.65
Mass (kg)	62.63 (10.52)	58.28	66.97	60.00
BMI (kg/m^2^)	22.07 (3.14)	20.77	23.37	21.40
IPAQ (MET/week)	4112.15 (3595.12)	2628.16	5596.15	2490.00

SD: standard deviation; BMI: body mass index; MET: metabolic equivalent of task; IPAQ: International Physical Activity Questionnaire.

**Table 2:** Comparison of muscle activation across different muscle groups, positions, and kinetic chains.

				Confidence Interval at 95%						
Muscle	Exercise	Chain	Mean (SD)	Lower Limit	Upper Limit	Median	Mean Differences	W	*p*	r
Left serratus anterior (µV)	Standing	Open-chain	87.73 (80.41)	54.60	120.9	70.91	−11.89	41	<0.001 **	−0.74
		Closed-chain	107.1 (92.65)	68.90	145.4	85.46				
	Kneeling	Open-chain	78.2 (49.16)	57.94	98.5	72.76	−14.50	9	<0.001 **	−0.94
		Closed-chain	100.0 (71.63)	70.42	129.6	81.04				
	Seated	Open-chain	93.9 (77.09)	62.07	125.7	76.07	−28.21	9	<0.001 **	−0.94
		Closed-chain	26.8 (15.13)	20.53	33.0	22.80				
Right serratus anterior (µV)	Standing	Open-chain	93.6 (60.82)	68.51	118.7	85.58	−16.47	2	<0.001 **	−0.98
		Closed-chain	72.7 (50.54)	51.84	93.6	63.95				
	Kneeling	Open-chain	74.2 (40.99)	57.31	91.1	68.56	−17.50	1	<0.001 **	−0.99
		Closed-chain	94.1 (48.74)	74.02	114.3	89.75				
	Seated	Open-chain	84.7 (58.92)	60.34	109.0	63.18	−26.90	19	<0.001 **	−0.88
		Closed-chain	115.2 (74.66)	84.35	146.0	88.20				
Left latissimus dorsi (µV)	Standing	Open-chain	27.2 (9.87)	23.16	31.3	25.95	−1.67	114	0.20	−0.29
		Closed-chain	29.1 (9.84)	25.03	33.2	28.70				
	Kneeling	Open-chain	26.0 (9.63)	22.07	30.0	24.43	−2.18	117	0.23	−0.28
		Closed-chain	28.2 (9.94)	24.05	32.3	26.21				
	Seated	Open-chain	30.0 (12.19)	24.97	35.0	26.07	−5.02	26	<0.001 **	−0.84
		Closed-chain	35.6 (11.07)	31.03	40.2	35.73				
Right latissimus dorsi (µV)	Standing	Open-chain	26.8 (11.65)	21.98	31.6	22.58	−2.07	99	0.09	−0.39
		Closed-chain	28.6 (10.99)	24.02	33.1	23.81				
	Kneeling	Open-chain	25.9 (9.48)	21.97	29.8	23.99	0.17	170	0.85	0.04
		Closed-chain	26.5 (11.31)	21.87	31.2	22.27				
	Seated	Open-chain	28.7 (10.76)	24.30	33.2	25.60	−5.60	20	<0.001 **	−0.87
		Closed-chain	35.2 (12.92)	29.84	40.5	30.69				

SD: standard deviation; µV: microvolts; W: Wilcoxon; *p*: significance index; r: effect size; **: *p* ≤ 0.01.

**Table 3:** Results of muscle activation differences between positions kinetic chain and the muscle.

Chain	Muscle	χ^2^	*p*
Open-chain	Left serratus anterior (µV)	1.42	0.31
	Right serratus anterior (µV)	2.96	0.22
	Left latissimus dorsi (µV)	8.24	0.01 *
	Right latissimus dorsi (µV)	5.36	0.06
Closed-chain	Left serratus anterior (µV)	7.44	0.02 *
	Right serratus anterior (µV)	10.3	0.006 **
	Left latissimus dorsi (µV)	27	<0.001 **
	Right latissimus dorsi (µV)	29.4	<0.001 **

µV: microvolts; χ^2^: Kruskal–Wallis; Friedman; *p*: significance index; *: *p* ≤ 0.05; **: *p* ≤ 0.01.

**Table 4:** Results of muscle activation differences between positions (standing, kneeling, and seated) and chain (open-chain and closed-chain).

Chain	Muscle	Exercises	DC	*p*
Open-chain	Left latissimus dorsi (µV)	Standing–Kneeling	1.06	0.29
		Standing–Seated	1.97	0.05
		Kneeling–Seated	3.03	0.004 **
Closed-chain	Left serratus anterior (µV)	Standing–Kneeling	0.45	0.65
		Standing–Seated	2.70	0.009 **
		Kneeling–Seated	2.25	0.02 *
	Right serratus anterior (µV)	Standing–Kneeling	0.46	0.643
		Standing–Seated	3.26	0.002 **
		Kneeling–Seated	2.80	0.007 *
	Left latissimus dorsi (µV)	Standing–Kneeling	1.02	0.313
		Standing–Seated	5.92	<0.001 *
		Kneeling–Seated	6.94	<0.001 *
	Right latissimus dorsi (µV)	Standing–Kneeling	0.86	0.393
		Standing–Seated	6.68	<0.001 *
		Kneeling–Seated	7.54	<0.001 *

µV: microvolts; DC: Durbin-Conover; *p*: significance index; *: *p* ≤ 0.05; **: *p* ≤ 0.01.

## Data Availability

The data presented in this study are available on request by the corresponding author.

## Supplementary Materials

The following supporting information can be downloaded at: https://www.mdpi.com/article/10.3390/muscles4030020/s1, Table S1: CEDE Checklist for reporting and critically appraising studies using EMG (CEDE-Check).

## Abbreviations

The following abbreviations are used in this manuscript:

EMG	Electromyography
HE	Hypopressive exercise
LD	Latissimus dorsi
MVIC	Maximal voluntary isometric contraction
SA	Serratus anterior
sEMG	Surface electromyography
