# Supplementary material for: Serratus Anterior and Latissimus Dorsi Muscle Activation in Hypopressive Exercises Performed in Open Versus Closed Kinetic Chain: A Cross-Sectional Study
Source: Muscles. 2025 Jun 23;4(3):20. doi: 10.3390/muscles4030020 (PMC12265994; doi:10.3390/muscles4030020)
Supplement: Supplementary file 1 [file muscles-04-00020-s001.zip › muscles-3674014-supplementary.pdf]

Supplementary file S1. CEDE Checklist for reporting and critically appraising studies using EMG (CEDE-Check)

Instructions: Mark each item on the checklist as either "reported", “not reported” or "not applicable" (N/A). If an item is reported, specify the page where the information can be found. If an item is not reported or partially reported, use the comments box to justify the decision. When completing the "Characteristics of recording electrodes" section, fill it out based on the type of electrode used. If a particular type of electrode isn't used, simply tick N/A and move on to the next section.

Section/topic	Item	Description	Reported	Not reported	N/A	Page	Comments
**Task(s)**							
	1	Context of research (e.g., laboratory, clinical setting, sport setting, daily-life environment)	☒	☐		5	
	2	Description of task(s) with sufficient detail for replication, should include description of the adopted set-up (e.g., start/end position, joint angles, body posture, or any other potential constraint) and equipment (if any)	☒	☐		5, 6, 7	
	3	Specify type of contraction (e.g., isometric, dynamic, concentric, eccentric) of the recorded muscle(s)	☒	☐		6	
	4	Specify duration/number of repetitions, recovery/rest period between repetitions/tasks, and speed of movement/contraction (or whether speed was defined by instructions like “self-paced”, “as fast as possible” or by a specific devices like treadmill, metronome, isokinetic machine)	☒	☐		6,7	
	5	Specify if part of the task/repetition(s)/trials(s) were not recorded or excluded, with justification for the removal of data.	☐	☐	NA		No part of the task/repetition(s) were not recorded or excluded. Therefore, there was no removal of data.
	6	Description of warm-up/familiarisation	☒	☐	☐		Familiarisation was included and described. The intervention did not include a warm-up due to the characteristics of the intervention protocol.

Section/topic	Item	Description	Reported	Not reported	N/A	Page	Comments
	7	Instructions given to the participant on how the task should be performed (e.g., "as fast as possible" or "as accurately as possible")	☒	☐	☐	6-7	
	8	When two or more tasks are performed, specify order or method of randomisation/counterbalancing	☒	☐	☐	7	
	9	If graded submaximal contractions are performed, specify how intensity was controlled (e.g., visual feedback from a screen), percentage of maximal voluntary contraction (MVC), instructions used, and how the MVC was assessed and calculated. Please refer to "CEDE Amplitude normalisation matrix" (Besomi et al., 2020).	☒	☐	☐	6	
	10	Description of task(s) performed for amplitude normalisation. Please refer to "CEDE Amplitude normalisation matrix" (Besomi et al., 2020))	☒	☐	☐	6	
**Procedure for EMG recording**							
**a) Electrode placement**	11	Muscle(s) investigated and side of body	☒	☐		5	
	12	Electrode type (e.g., conventional surface electrode, array or grid of electrodes, fine-wire electrode, needle electrode). Please refer to the "CEDE Electrode selection matrix" (Besomi et al., 2019)	☒	☐		5	
	13	Procedure for skin preparation (both for surface and intramuscular electrodes). Describe steps of the procedure (e.g., shaving of hair, scrubbing of skin, antibacterial skin preparation) and products used (e.g., abrasive paste including manufacturer).	☒	☐		6	
	14	Description of electrode placement with sufficient detail for replication and reference to guidelines as appropriate (e.g., SENIAM, CEDE (Besomi et al., 2020; Besomi et al., 2019; Gallina et al., 2022; Martinez-Valdes et al., 2023; McManus et al., 2021), Journal of Electromyography and Kinesiology tutorials (Clancy et al., 2023; Del Vecchio et al.,	☒	☐		6	SENIAM guidelines for electrode placement and location were followed.

Section/topic	Item	Description	Reported	Not reported	N/A	Page	Comments
		2020; Merletti & Cerone, 2020; Merletti & Muceli, 2019)). Provide details on the adopted methods to ensure appropriate electrode placement and orientation with respect to muscle fascicle direction (e.g., along muscle fibres, distance from anatomical landmarks, ultrasound guided) depending on study aim and feasibility for the investigated muscle. If electrode placement differs from guidelines, specify why, and describe in detail.					
	15	Positioning of reference electrode(s).	☒	☐	☐	6	
	16	If multiple sessions are performed with the intention to record from a similar region of a muscle, describe the methods adopted to ensure consistency of electrode placement.	☐	☐	☒		
**b) Characteristics of recording electrodes**	*Surface electrodes (including reference electrode(s))*		☒	☐	☐	5, 6	
	17	Physical configuration (e.g., concentric, bipolar, array, grid)	☒	☐		5	
	18	Electrode size (e.g., diameter of recording area of the electrode, length), material (e.g., Ag/AgCl), and shape (e.g., circular, bar). If determined by a company/manufacturer, report this from the information provided.	☒	☐		5	
	19	Interelectrode distance (specify center to center, or edge to edge). If determined by a company/manufacturer, report this from the information provided.	☒	☐		6	
	20	Number of electrodes (if electrode grid was used, specify the number of rows and columns, and missing electrode(s) if any)	☒	☐		5-6	
	21	Electrode type (wet, dry, insulating), model and company (if appropriate), characteristics of the electrode skin contact (e.g., double adhesive foam, adhesive tape, Velcro belt, use of conductive paste or gel) and electrode fixation.	☒	☐		6	
	Fine wire intramuscular electrodes		☐	☐	☒		

Section/topic	Item	Description	Reported	Not reported	N/A	Page	Comments
	22	Recording montage (e.g., bipolar, monopolar, others)	☐	☐			
	23	Wire type and properties (e.g., diameter, wire, and insulation material, single or multistrand, characteristics of the conductive wire, method for insulation removal)	☐	☐			
	24	Approximate length of exposed conductor and bent tips. If determined by a company/manufacturer, report this from the information provided.	☐	☐			
	25	For a bipolar pair: separation between electrodes and how this was controlled (glued pair, staggered pair, monopolar with respect to a surface reference). If determined by a company/manufacturer, report this from the information provided.	☐	☐			
	26	Size (diameter and length) of the needle used for insertion, orientation during insertion and fine wire fixation technique (if any)	☐	☐			
	Needle intramuscular electrodes		☐	☐	☒		
	27	Type of needle (e.g., monopolar, concentric, bipolar, quadrifilar, tungsten) including brand	☐	☐			
	28	Needle size (gauge), length, and type of metal contact (e.g., stainless steel)	☐	☐			
	29	Describe the orientation during insertion	☐	☐			
**c) Acquisition of EMG signals and pre-processing**	30	Detection mode of EMG signals (e.g., monopolar, single differential, double differential, etc)	☐	☐			
	31	Brand and model of the EMG acquisition system, or report details (i.e., input impedance, equivalent input voltage noise, bandwidth, common mode rejection ratio) if custom-built.	☐	☐			
	32	Gain of amplifier and cut-off frequencies of hardware filter (and if possible, filter type and order). If determined by a	☐	☐			

Section/topic	Item	Description	Reported	Not reported	N/A	Page	Comments
		company/manufacturer, report this from the information provided.					
	33	Sampling frequency (Hertz or samples/s). If determined by a company/manufacturer, report this from the information provided.	☐	☐			
	34	Analog-to-digital (A/D) resolution (bits) and full range (without risk of saturation). If determined by a company/manufacturer, report this from the information provided.	☐	☐			
	35	Name and version of the software used to record the EMG signals or specify if custom-made.	☐	☐			
	36	Technique(s) applied for power line interference removal (none, driven right leg (DRL), notch filter(s), frequency interpolation, others), including the relevant features (width of band reject, filter order, number of power line interference harmonics removed, etc.)	☐	☐	☐	6	
	37	Acquisition/synchronisation with other devices (when distinct channels are not automatically synchronized. e.g., force, movement capture). Report any inherent delay (if any) induced by the recording system, and how it was compensated for.	☒	☐	☐		
	38	If wireless, specify method of transmission (e.g., Bluetooth, Radiofrequency)	☒	☐	☐	6	
	39	If wearable system was used, specify the dimension, weight, fixation method, and transmission range. If determined by a company/manufacturer, report this from the information provided.	☒	☐	☐	6	
	40	Pre-amplification of signal and location relative to the electrode (if used)	☒	☐	☐		5
